# ‘Dirty dose’‐based proton variable RBE models ‐ performance assessment on in vitro data

**DOI:** 10.1002/mp.17519

**Published:** 2024-11-20

**Authors:** Fredrik Kalholm, Iuliana Toma‐Dasu, Erik Traneus

**Affiliations:** ^1^ Medical Radiation Physics Department of Physics Stockholm University Stockholm Sweden; ^2^ Department of Oncology and Pathology Medical Radiation Physics Karolinska Institutet Stockholm Sweden; ^3^ Raysearch Laboratories AB Stockholm Sweden

**Keywords:** dirty dose, LET, RBE

## Abstract

**Background:**

In clinical proton radiotherapy, a constant relative biological effectiveness (RBE) of 1.1 is typically applied. Due to abundant evidence of variable RBE effects from in vitro data, multiple variable RBE models have been suggested, typically by describing the α and β parameters in the linear quadratic (LQ) model as a function of dose averaged linear energy transfer (${\text{LET}}_{d}$).

**Purpose:**

This work introduces a new variable RBE model based on the dirty dose concept, where dose deposited in voxels with a corresponding LET exceeding a specific threshold is considered “dirty” in the sense that it has a biological effect above the one predicted by a constant RBE of 1.1. As only one LET level, corresponding to a specific energy for a given particle in a given medium, needs to be monitored, this offers several advantages, such as simplified calculations by removing the need for intricate end of range LET calculations and averaging procedures, as well as opening up for more efficient experimental assessment of the cell specific model parameters.

**Methods:**

Previously published in vitro data were utilized, where surviving fraction (SF), dose and ${\text{LET}}_{d}$ were reported for a pristine proton beam with varying physical PMMA thicknesses placed upstream of the cells. The setup was re‐simulated to extract dirty dose metrics for the corresponding reported ${\text{LET}}_{d}$‐values. Models were created by setting the α parameter of the LQ model as a function of the fraction of dirty dose and subsequently benchmarked against models based on other radiation quality metrics by comparing the root‐mean‐square‐error (RMSE) of the predicted and actual cell surviving fraction.

**Results:**

Variable RBE models based on dirty dose perform on par with conventional radiation quality metrics with a RMSE of 0.38 for a dirty dose‐based model with a threshold of 7 keV/μm, compared to 0.42 and 0.36 for a ${\text{LET}}_{d}$‐based and $Q_{\mathrm{eff},d}$‐based model, respectively. Higher chosen LET thresholds typically performed better and lower performed worse.

**Conclusion:**

The results indicate that models based on dirty dose metrics perform equally well as conventional radiation quality metrics. Due to the simplified calculations involved and the potential for more efficient measurement techniques for data generation, dirty dose‐based models might be the most conservative and practical approach for creating future proton variable RBE models.

## 1 INTRODUCTION

In clinical proton radiotherapy, a fixed relative biological effectiveness (RBE) of 1.1 is conventionally employed, implying that protons are consistently 10% more effective at inactivating cells compared to photons, irrespective of proton energy and cell type.^1^ Although this assumption of constant RBE has yielded satisfactory clinical outcomes, numerous studies based on both in vitro data^2^ and analysis of patient outcomes^3^, ^4^, ^5^ suggest that the RBE actually varies with proton energy. However, there is no consensus on how to quantify this effect using a specific variable RBE model. In clinical practice, the concern associated with RBE variability is therefore usually addressed by reducing end of range dose, where the variable RBE is presumed to be highest and also most uncertain, near distal risk organs.

Historically, various variable RBE models have been proposed, primarily derived from in vitro data. A common approach involves describing the α and β parameters of the linear quadratic (LQ) model as a function of a chosen radiation quality metric.^2^ Typically, this metric entails an averaged value of linear energy transfer (LET).^1^ with dose‐averaged ${\text{LET}}_{d}$ ^1^ being the most prevalent.^6^, ^7^, ^8^, ^9^, ^10^, ^11^, ^12^ The ${\text{LET}}_{d}$ including all protons and reported in 1.0 $g/{\mathrm{cm}}^{3}$ water, has emerged as the most common radiation quality metric across many European proton treatment facilities,^13^ a welcoming initiative following a scattered reporting throughout the literature.^14^

Other proposed radiation quality metrics include dose averaged Q ($Q_{d}$), Q being defined as $Z^{2}/E$ where Z and E being the charge and kinetic energy of an ion^15^ and dose averaged $Q_{\mathrm{eff}}$ ($Q_{\mathrm{eff},d}$), $Q_{\mathrm{eff}}$ being defined as $z^{*2}/\beta^{2}$ where $z^{*2}$ denotes the effective charge and β denotes the speed relative to light in vacuum. $Q_{\mathrm{eff}}$ can be shown to be proportional to the number of electrons emitted per track length.^16^ Since LET is proportional to the total energy released per path length, higher energy electrons have a higher weight associated in comparison to $Q_{\mathrm{eff}}$, despite potentially depositing a significant portion of their energy far from the primary track, which may not contribute to an increased RBE at the point of calculation. A recent study have demonstrated that both $Q_{d}$ and $Q_{\mathrm{eff},d}$ outperform ${\text{LET}}_{d}$ as radiation quality metrics for variable RBE models^17^

Another quantifiable approach to address the presumed variable RBE dose in proton therapy is by monitoring the energy deposited over a specific LET threshold value, known as ‘dirty dose’.^18^ To clarify, this should not be confused with energy deposited in voxels above a specific *averaged* LET value, but rather dose deposited above a given LET value per each individual particle entering the voxel. The vast majority of the dirty dose stems from stopping protons, but in the entry region of the beam, a significant part will also come from 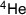 particles, created by nuclear reactions from high energy protons. Integrating this concept into the planning optimization process allows for minimizing the dirty dose to organs at risk (OAR),^19^ serving as an additional degree of freedom in the planning process. In analogy with application on OARs, one can maximize the dirty dose in the target volume. Similarly, a constraint can be imposed on proton track‐ends, aiming to minimize their presence in organs at risk given their association with high LET values. For carbon ion therapy, dirty dose has also been suggested as a useful parameter for target evaluation.^20^ Utilizing dirty dose or the number of track‐ends as radiation quality metrics for a variable RBE model offers advantages over traditional averaged LET (or $Q_{\mathrm{eff}}$) based metrics. The dirty dose or track‐end based metrics are straightforward to compute, requiring no averaging across incident particle fluence, which is particularly advantageous in clinical plans with overlapping beams with large spread of LET inside voxels and between voxels.

A recent study proposed a variable RBE model, estimating RBE as a function of the number of proton track‐ends.^21^

This work introduces a novel variable RBE model based on dirty dose, deriving a LQ model based formalism for it, and attempts to benchmark it against ${\text{LET}}_{d}$ and $Q_{\mathrm{eff},d}$ based models. Additionally, we propose a new measurement methodology to characterize cell line‐specific model parameters, enabling the use of the proposed RBE model in clinical treatment planning.

## 2 METHODS

### 2.1 Theory

#### 2.1.1 Model motivation

In clinical treatment planning, relevant quantities are, most commonly, computed and reported in a cartesian three‐dimensional grid of point values. The reported value per point represents the quantity considered in some macroscopic volume containing the point. Typically, that volume is a cube with dimensions of a few millimeters that is, the “voxel”. The above applies, for example, to physical dose, RBE‐weighted dose, and ${\text{LET}}_{d}$. The RBE‐weighted dose, in particular, represents the average RBE‐weighted dose in a dose voxel and the RBE factor represents the average RBE in the same volume. In this work, we will refer to such a volume as a “macroscopic” volume and we seek a sound method to model the proton RBE in this macroscopic volume.

In the literature, the vast majority of RBE models considered for proton treatment planning have as the starting point in vitro cell line clonogenic survival assay data.^2^ In so called *microdosimetric* RBE models, such as the local effect model^22^ or the microdosimetric kinetic model^23^ mostly used for helium and carbon therapy, LQ model dose averaged α and β are computed via microdosimetry based track structure characteristics constrained by parameters fitted to experimental survival data with dose averaged evaluation of α and β as proposed by Rossi‐Zaider.^24^

Another class of RBE models are those where LQ model α and β are fitted directly to survival data as function of a chosen irradiation quality metric. For instance, in the widely used McNamara model,^10^ the RBE factor is computed from ${\text{LET}}_{d}$ (together with dose and the value of photon $\alpha_{x}/\beta_{x}$, x denoting irradiation by photons, via the LQ model) and four model parameters determined from fits of a set of dose and dose averaged LET data points. The same is true also for the Guan et al. data^25^, ^26^, ^27^ used in this work. One can consider this type of models to be quasi‐microdosimetric as the RBE factor can, in fact, be applied per proton track if the fraction dose is known per voxel or one can apply the Rossi‐Zaider averaging.

RBE factor calculations by microdosimetric or quasi‐microdosimetric LQ based models as described above can be well justified from a conceptual point of view in idealized situations. Its application in treatment planning is, however, problematic from a practical point of view, which is one of several factors hampering clinical introduction of variable RBE in proton planning. Accurate clonogenic cell line survival measurements at low energies (high LET) are not straightforward. Most often, plated cells are irradiated by beams where the cells are exposed to a range of relatively broad and overlapping LET spectra where each spectrum represents a nominal irradiation quality point (see, e.g., Guan et al.^25^ where data are reported for a set of ${\text{LET}}_{d}$ values where the LET spectra are unique for the measurement setup at that experiment). Extracting α and β values as a function of LET from such data requires a meticulous beam line characterization and LET spectrum deconvolution procedure. Applying an unrealistic linear RBE(LET) relationship is mandated, that is, by using ${\text{LET}}_{d}$ as a radiation quality metric. Tabulating survival data in order to extract α and β as a function of (nominal) averaged LET over a wide range of LET and dose values, means a large number of sufficiently accurate survival data points must be acquired. Further, for clinical planning knowledge of cell line specifics on a *microscopic* level as defined above should not, after all, be a necessary prerequisite. The end result in all calculations relevant for treatment planning is anyway RBE‐weighted dose averaged over a *macroscopic* sized dose voxel volume where the RBE factor arises from an integration of biological effect over the particle fluence in the voxel; the underlying microscopic features are not subject for any treatment plan considerations. This circumstance is one driving motivation for developing the new RBE model presented in this work. The model is tailored for application in voxelized geometries as used in treatment planning where relevant volumes are 1 – 3 mm cubes which we here refer to as a macroscopic volume.

In the next subsection, we introduce the basic concepts of the new model, named dirty dose kill model, or DDK for short. The following subsection elaborates on the DDK model formalism and its application to voxelated calculations as performed in treatment planning. In the discussion section of this paper, we outline how cell line specific DDK model parameters can potentially be determined from only very few cell survival data points.

#### 2.1.2 Model foundation and its application in the LQ formalism

The DDK model posits that the increased biological effects occur near the end of the protons' range. Accordingly, we assume that there exists an LET threshold below which the biological effect of proton energy depositions is the same as the effect of photons that is, RBE = 1. For application in treatment planning calculations, we divide a plan's physical dose distribution** in each voxel in two compartments: dirty dose and clean dose, denoted by $D_{D}$ and $D_{C}$, respectively, see Heuschel et al. ^18^. The dirty dose $D_{D}$ arises from proton energy depositions above a certain LET threshold ${\mathrm{LET}}_{\mathrm{DD}}$, and clean dose $D_{C}$ arises from energy depositions below ${\mathrm{LET}}_{\mathrm{DD}}$. The sum of the dirty dose and the clean dose equals the total dose per voxel:

(1) $$D=D_{D}+D_{C}$$

In the following, lets first consider a single LET threshold denoted by ${\mathrm{LET}}_{\mathrm{DD}}$. The LQ model for cell survival stipulates that:

(2) $$-\ln\left(S\right)=\alpha D+\beta D^{2}$$

where ln(S) is the natural logarithm of survival, α and β are cell line specific parameters and D is the fraction dose for the considered irradiation quality. For two irradiation qualities resulting in the same surviving fraction, where one is a photon reference quality and the other is a proton quality, it follows that:

(3) $$\alpha_{x}RD+\beta_{x}R^{2}D^{2}=\alpha D+\beta D^{2}$$

where $\alpha_{x}$ and $\beta_{x}$ are the reference quality cell line parameters, α and β are corresponding quantities for the proton quality, D is the proton fraction dose and R is shorthand for RBE. For this to hold over a range of doses, and in a LQ model context where we consider proton α and β to be independent of fraction dose, we must have:

(4) $$\alpha_{x}RD=\alpha D\;\;\;\;\mathrm{and}\;\;\;\;\beta_{x}R^{2}D^{2}=\beta D^{2}$$

For the part linear with dose, and introducing the dirty dose and clean dose, we get:

(5) $$\alpha_{x}RD=\alpha \left(D_{C}+D_{D}\right)$$

or

(6) $$\alpha_{x}RD=\alpha_{x}\left(1-f\right)D+\alpha_{D}fD$$

where we introduced the dirty dose fraction $f=D_{D}/D$, denoting by $\alpha_{D}$ the α of the dirty dose compartment, and in addition applied $\alpha_{x}$ acting on the clean dose compartment, which in this is model is assumed to be photon like. Finally, we introduce the cell line specific parameter ξ defined by:

(7) $$\alpha_{D}=\alpha_{x}\left(1+\xi \right)$$

Note that in Equation 7, the quantity ξ should be interpreted as a cell line specific natural constant similar to for example, $\alpha_{x}$ and not a model fitting parameter. Rearranging we get:

(8) $$\alpha_{x}RD=\alpha_{x}\left(1+f\xi \right)D$$

from which we can conclude:

(9) $${\mathrm{RBE}}_{\max}=1+f\xi$$

where ${\mathrm{RBE}}_{\max}$ is, per definition, RBE in the limit of low dose for a voxel with dirty dose fraction f.

This final result of Equation 9 is striking in that for ${\text{RBE}}_{\max}$, all cell line radiobiological effects for the end point considered, accumulated over the entire stopping down spectrum from the energy defined by ${\mathrm{LET}}_{\mathrm{DD}}$ down to zero energy, are contained in the single parameter ξ. As will be speculated in the discussion part, ξ is accessible for direct measurement provided certain experimental conditions can be created.

It should be stated that the expressions introduced in this section are not directly applicable to treatment planning for a general case with arbitrary voxel sizes and composite fields where each voxel senses a voxel specific and typically broad spectrum of LET values. Thus, the next section will further elaborate, starting from the model foundation presented in this section, with the goal of obtaining a practical, usable formulation of the DDK model.

**FIGURE 1:**
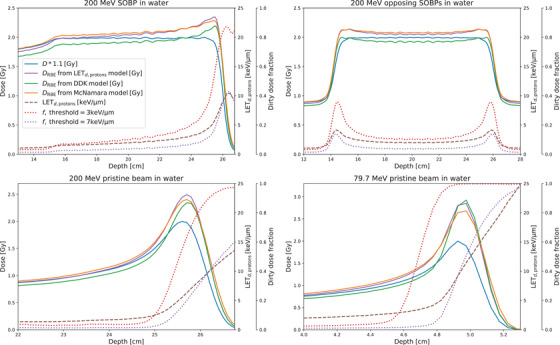
Shows RBE‐doses from different models (solid lines, with the DDK model using a LET threshold of 7 keV/μm), dirty dose fractions (dotted lines) and ${\text{LET}}_{d,\mathrm{protons}}$‐values (dashed lines) for a 200 MeV SOBP (upper left panel), two opposing 200 MeV SOBP's (upper right panel), a 200 MeV pristine beam (lower left panel) as well as for a 79.7 MeV pristine beam. The latter corresponds to the in vitro experimental setup used for the models developed in this work. DDK, dirty dose kill; RBE, relative biological effectiveness.

In Figure 1, the dirty dose fraction is illustrated for a mono‐energetic proton integrated depth dose (IDD) and a proton spread out bragg peak (SOBP) for two LET thresholds: ${\mathrm{LET}}_{\mathrm{DD}}$= 3 keV/μm and ${\mathrm{LET}}_{\mathrm{DD}}$= 7 keV/μm corresponding to 16.8 MeV and 5.8 MeV proton energies, respectively, with residual ranges in unit density water of 3.1 and 0.5 mm, respectively.^28^

#### 2.1.3 Model elaboration

Starting from Equation 9 in the previous section, we generalize to circumstances relevant for clinical treatment planning with typical voxel sizes from 1 to 3 mm and multiple overlapping beams. A low LET threshold and a high LET threshold are introduced, denoted by ${\mathrm{LET}}_{\mathrm{DD},\mathrm{low}}$ and ${\mathrm{LET}}_{\mathrm{DD},\mathrm{high}}$, respectively. Two additional parameters to carry cell line specificity (RBE at LET < ${\mathrm{LET}}_{\mathrm{DD},\mathrm{low}}$ and RBE at LET > ${\mathrm{LET}}_{\mathrm{DD},\mathrm{high}}$) while being largely decoupled from plan characteristics such as voxel size and variations of LET spectrum between voxels are also introduced.

Firstly, recognizing that the overarching posit of the DDK model that biology near end of range is completely predictive of RBE is too simplistic, we allow RBE to be different from unity, but constant, if LET < ${\mathrm{LET}}_{\mathrm{DD},\mathrm{low}}$ (i.e., the clean dose compartment) by introducing the parameter $R_{0}$ in equations 6 to 8 yielding

(10) $$\alpha_{x}RD=\alpha_{x}R_{0}\left(1-f\right)D+\alpha_{x}\left(1+\xi \right)fD$$

The second addition is more intricate and is introduced to account for variation of the LET spectrum between voxels. In this clinically relevant situation, the parameter ξ alone will not reflect the average ${\text{RBE}}_{\max}$ factor in a voxel, as the stopping down spectrum of protons entering a voxel is not the same, due to a plethora of reasons (energy straggling, overlapping beams, etc.). This circumstance is obvious from the dirty dose fractions for the low LET and high LET thresholds, from hereon denoted by $f_{\mathrm{low}}\;\mathrm{LET}$ and $f_{\mathrm{high}}\;\mathrm{LET}$, respectively, shown in Figure 1 for an integrated depth dose (IDD). For the low LET threshold, $f_{\mathrm{low}}\;\mathrm{LET}$ saturates to unity when approaching the distal part of the IDD as the amount of protons stopping in a voxel increases. This is reflected in the LET spectrum and thereby, in the amount of dirty dose in the voxel. The high LET dirty dose fraction, $f_{\mathrm{high}}\;\mathrm{LET}$, shows the same trend but does not reach saturation.

In the following, we will define a set of helper quantities to support the formalism. We denote by Γ the ratio of the dirty doses at the low LET and high LET thresholds in a voxel:

(11) $$\Gamma =\frac{\mathrm{Dirty}\;\mathrm{dose}\;\mathrm{at}\;\mathrm{high}\;\mathrm{LET}\;\mathrm{threshold}}{\mathrm{Dirty}\;\mathrm{dose}\;\mathrm{at}\;\mathrm{low}\;\mathrm{LET}\;\mathrm{threshold}}$$

We define an ideal irradiation condition, denoted from hereon as the DDK condition, where a voxel only receives protons that enter the voxel at exactly energy $E_{\mathrm{low}}\;\mathrm{LET}$ and then come to rest in the voxel. Hence, under DDK conditions and from the definition of dirty dose, it follows that

(12) $$\Gamma_{\mathrm{DDK}}=\frac{E_{\mathrm{high}}\;\mathrm{LET}}{E_{\mathrm{low}}\;\mathrm{LET}}$$

For the exemplary numbers discussed above we have

(13) $$\Gamma_{\mathrm{DDK}}=\frac{E_{7\mathrm{keV}/\mu m}}{E_{3\mathrm{keV}/\mu m}}=\frac{5.8\mathrm{MeV}}{16.8\mathrm{MeV}}=0.35$$

For an arbitrary voxel, where the DDK condition is generally not fulfilled, we define a corresponding ratio $\Gamma_{Q}$ which can be computed as:

(14) $$\Gamma_{Q}=\frac{f_{\mathrm{high}}\;\mathrm{LET}}{f_{\mathrm{low}}\;\mathrm{LET}}$$

The suffix Q indicates that $\Gamma_{Q}$ should be considered as a voxel specific metric of “LET quality”; if the value of $\Gamma_{Q}$ is high, the LET spectrum is skewed in the high LET direction, and vice versa if the value of $\Gamma_{Q}$ is low. Finally, we will apply the ratio

(15) $$Q_{\mathrm{LET}}=\frac{\Gamma_{Q}}{\Gamma_{\mathrm{DDK}}}$$

as the metric for a voxel's LET quality relative of a voxel irradiated under the ideal DDK condition. Note that the value of $\Gamma_{Q}$ lies in the interval [0,1] whereas the value of $Q_{\mathrm{LET}}$ lies in the interval [0, 1/$\Gamma_{\mathrm{DDK}}$]. We will use $Q_{\mathrm{LET}}$ to correct, in the simplest possible manner and to first order, for the variable presence of stopping protons and thereby deviating from the DDK condition.

In the following subsections we present two equivalent formulations of the DDK model. The first is applicable if a direct measurement of ξ under DDK condition is available or, alternatively, when α as a function of LET, i.e the function α(LET), is known a priori in which case ξ can be calculated. Note that the latter is rarely the case experimentally, and is, in fact, often the ultimate goal in radiobiological characterizations of cell radiation responses. This formulation is obviously suitable in a situation where the α(LET) function is known from an independent model or from adequately processed experimental data. The second formulation is applicable in the case where there exists experimental data which have been acquired without the DDK condition being fulfilled. We demonstrate that it is possible to determine the cell line specific DDK parameters from such data, which allows RBE to be evaluated for any irradiation situation.

#### 2.1.4 Application 1: Fitting to measurements under DDK condition

Under DDK conditions, we must have

(16) $$1+\xi =\frac{\int_{0}^{E_{\mathrm{low}}\;\mathrm{LET}}\alpha \left(\mathrm{LET}\left(E\right)\right)dE}{\alpha_{x}E_{\mathrm{low}}\;\mathrm{LET}}=R_{1}\left(1-\Gamma_{\mathrm{DDK}}\right)+R_{2}\Gamma_{\mathrm{DDK}}$$

where α(LET(E)) denotes the (unknown) function that gives α of the cell line for a given LET. In Equation 16, $R_{1}$ and $R_{2}$ are RBE‐values, defined by dividing the integral into two intervals: from $E_{\mathrm{low}}\;\mathrm{LET}$ to $E_{\mathrm{high}}\;\mathrm{LET}$ and from $E_{\mathrm{high}}\;\mathrm{LET}$ to E=0 with

(17) $$R_{2}=1+\xi^{\prime }=\frac{1}{\alpha_{x}E_{\mathrm{high}}\;\mathrm{LET}}\int_{0}^{E_{\mathrm{high}}\;\mathrm{LET}}\alpha \left(\mathrm{LET}\left(E\right)\right)dE$$

Solving for $R_{1}$ we get

(18) $$R_{1}\left(\xi ,R_{2}\right)=\frac{\left(1+\xi \right)-R_{2}\Gamma_{\mathrm{DDK}}}{1-\Gamma_{\mathrm{DDK}}}$$

The quantity $R_{2}=1+\xi^{\prime }$ is analogous to 1+ξ of Equation 7, but valid for the high LET threshold. Rearranging equation 10, we can write

(19) $$\alpha_{x}RD=\alpha_{x}\left(R_{0}\left(1-f\right)+R_{1}\left(1-\Gamma_{\mathrm{DDK}}\right)f+R_{2}\Gamma_{\mathrm{DDK}}f\right)D$$

Finally, we apply the LET quality factor $Q_{\mathrm{LET}}$ defined in equation 15, adjusting the distribution of dirty dose between $R_{1}$ and $R_{2}$ according to the deviation from the DDK‐condition, resulting in

(20) $$\begin{array}{ccc} \alpha_{x}RD & = & \alpha_{x}(R_{0}\left(1-f\right)+R_{1}\left(1-\Gamma_{\mathrm{DDK}}Q_{\mathrm{LET}}\right)f\\ & & +R_{2}\Gamma_{\mathrm{DDK}}Q_{\mathrm{LET}}f)D \end{array}$$

It should be noted that the voxel specific LET quality factor $Q_{\mathrm{LET}}$ as introduced here is as a purely empirical and geometry driven correction for deviation from the ideal DDK condition. Here we apply it in the simplest possible manner as a linear scaling of the dirty dose factor in the voxel.

Equation 20 is applicable in a scenario where there exists independent measurements of RBE at low LET (i.e., parameter $R_{0}$), a measurement of ξ, and a measurement of at least one survival data point where $Q_{\mathrm{LET}}>1$. This allows the value of $R_{2}$ to be determined. Alternatively, the value of $R_{2}=1+\xi^{\prime }$ can be calculated from Equation 17 if α(LET) is known or be measured similar to how ξ can be measured.

If measurements are set up so that we have two macroscopic volumes, the thickness of which corresponds to the residual range of protons of the ${\mathrm{LET}}_{\mathrm{DD},\mathrm{low}}$ and ${\mathrm{LET}}_{\mathrm{DD},\mathrm{high}}$ thresholds, the ξ and $\xi^{\prime }$ parameters can be estimated for a given cell line using only two cell survival data points. Instead of attempting to estimate an RBE(LET) relationship across a wide range of LET values, requiring a corresponding wide range of measurements, the ξ and $\xi^{\prime }$ parameters contain the average RBE(LET) across a range of LET values simultaneously. As the average RBE value in a macroscopic volume is what ultimately will be evaluated for a clinical treatment plan voxel, the underlying exact RBE(LET) matters less if the average RBE can be well estimated in a given high LET range, corresponding to‐end‐of range protons.

#### 2.1.5 Application 2: Fitting to measured survival versus dose and LET spectrum data set

Here the starting point is a classic clonogenic cell survival assay data set with survival versus dose and irradiation quality data pairs as in for example, refs.[25, 26, 27]. As discussed in Section 2.1.1, the irradiation quality is rarely as simple as a mono‐energetic energy beam (in which case the model formulation of Section 2.1.4 is better suited). More common is that the irradiation quality is unique per reported data set. In the following, we simply assume we can reproduce the irradiation quality by simulation.

As in the previous section, we make use of a lower LET threshold and a higher LET threshold. From detailed specifications of the experimental setup the corresponding dirty and clean dose fractions can be computed per data point. The DDK modeling ansatz is then to compute RBE according to

(21) $$\begin{array}{ccc} & & \alpha_{x}RD=\alpha_{x}D\\ & & \left(R_{0}\left(1-f_{\mathrm{low}}\;\mathrm{LET}\right)+R_{1}\left(f_{\mathrm{low}}\;\mathrm{LET}-f_{\mathrm{high}}\;\mathrm{LET}\right)+R_{2}f_{\mathrm{high}}\;\mathrm{LET}\right) \end{array}$$

and fit values of $R_{0}$, $R_{1}$, and $R_{2}$ which represent the RBE values for clean dose, dirty dose below the high LET threshold and dirty dose above the high LET threshold, respectively. Furthermore, in line with many other publications,^6^, ^7^, ^9^, ^11^, ^29^, ^30^ we will assume that the increased lethality of proton irradiation stems from an increased number of single hit‐kills, that is, that all the increased RBE stems from a larger α value. Therefore, we set the proton β value to be equal to the photon value $\beta_{x}$.

As variable RBE values are expected to vary with α and β values of different cell lines, non‐cell line specific parameters ${\hat{R}}_{D}$, ${\hat{R}}_{1}$ and ${\hat{R}}_{2}$ are finally introduced, which will become the fitting parameter of the proposed RBE models. In this work, we assume an inverse relationship with RBE and the $\alpha_{x}/\beta_{x}$ value of the irradiated cell line, in line with many other publications,^7^, ^8^, ^9^, ^10^, ^11^, ^12^, ^30^ resulting in:

(22) $$R_{D}=\frac{{\hat{R}}_{D}}{\alpha_{x}/\beta_{x}},\;R_{1}=\frac{{\hat{R}}_{1}}{\alpha_{x}/\beta_{x}}\;\mathrm{and}\;R_{2}=\frac{{\hat{R}}_{2}}{\alpha_{x}/\beta_{x}}$$

where ${\hat{R}}_{D}$ becomes the fitting parameter for a single dirty dose threshold model, based on Equation 3, while ${\hat{R}}_{1}$ and ${\hat{R}}_{2}$ becomes the fitting parameter from a dual threshold model based on Equation 21.

In summary, when fitting to proton data or models there are two or three independent parameters, depending on the number of thresholds applies: $R_{0}$ and $R_{D}$ or $R_{0}$, $R_{1}$ and $R_{2}$, for a single or dual dirty dose threshold model, respectively, where the following should hold:

(23) $$R_{0}\leq R_{D}\;\mathrm{and}\;R_{0}\leq R_{1}\leq R_{2}$$

For heavier ions where an overkill effect (i.e., a decrease in RBE) can be expected with higher LET values, these relationships will no longer hold.

We conclude this section by emphasizing that the virtue of the proposed DDK based LQ formalism is that it is possible to determine, from data sets measured under unique experimental conditions, cell line specific parameters that allow RBE to be evaluated for arbitrary irradiation conditions.

### 2.2 Experimental set‐up

The data utilized in this study was published in three separate publications by MD Anderson and GSI based research groups.^25^, ^26^, ^27^ Briefly, it consists of H460 and H1437 cells being irradiated in vitro with a 79.7 MeV pristine pencil scanning proton beam on an in‐house jig built from PMMA, shown in figure 1f in Guan et al.^25^ This jig is designed to allow for 12 different thicknesses of PMMA, corresponding to 12 different averaged LET values (specifically, ${\text{LET}}_{d}$ of the primary protons is reported), with 8 repetitions of each thickness, resulting in 12 × 8  =  96 cell samples per irradiation.

By also changing the dose delivered in different irradiations, the data set includes both variation in ${\text{LET}}_{d}$ (ranging from 0.9 to 21.4 keV/um) and dose (ranging from 0.04 to 9.1 Gy), allowing for modeling with respect to both dose and beam quality to be performed. In total, by slightly adjusting the jig for the different publications, 24 different ${\text{LET}}_{d}$ values are reported, each consisting of 8‐64 repetitions for each dose, resulting in a total of 474 individual dose, cell and ${\text{LET}}_{d}$ combinations.

### 2.3 Reconstruction of experimental set‐up by simulation

To obtain the various quantities for building the RBE models, Raystation (version 11B‐IonPG) was utilized. This version has the same proton Monte Carlo transport algorithm as in the clinical version. The ${\text{LET}}_{d}$ and $Q_{\mathrm{eff},d}$ were scored by numerical integration over the stopping down spectrum at each voxel traversal. For protons stopping in the voxel or exiting the voxel with an energy below 16 MeV, the energy loss is divided into equidistant logarithmic steps with a step defined by requiring 90 steps between 20 keV and 16 MeV. For each step, the stopping power or $Q_{\mathrm{eff}}$, is computed and accumulated. For higher energies a two point formula is used for the integration. For ${\text{LET}}_{d}$, this procedure yields near identical results to the clinical version of RayStation where the integration is pre‐tabulated. For a given LET/energy threshold $E_{\mathrm{threshold}}$, dirty dose is trivial to score. For example, consider a proton that enters and exits a voxel at energies $E_{1}$ and $E_{2}$, respectively. If $E_{2}$ > $E_{\mathrm{threshold}}$ all deposited energy yields a clean dose. If $E_{1}$ < $E_{\mathrm{threshold}}$ all deposited energy yields a dirty dose. In the intermediate case, the clean and dirty dose contributions are given by $E_{1}$ ‐ $E_{\mathrm{threshold}}$ and $E_{\mathrm{threshold}}$ ‐ $E_{2}$, respectively.

A simulated block of PMMA was irradiated by a 79.7 MeV pristine pencil scanning proton beam using 0.5 mm voxels, broad enough to achieve lateral charged particle equilibrium. For each voxel depth, the ${\text{LET}}_{d,\mathrm{primary}}$ value for water (symbolizing the cell media) was calculated. These different ${\text{LET}}_{d}$ values are subsequently interpolated by cubic splines to obtain the corresponding physical PMMA thickness for each reported experimental ${\text{LET}}_{d}$ value.

After calculating the different depths, other radiation quality metrics corresponding to a specific ${\text{LET}}_{d,\mathrm{primary}}$ value can similarly be estimated by scoring an average value at each voxel depth and then interpolating quadratically to estimate the value at the calculated PMMA depth. The radiation quality metrics scored consisted of three different dirty dose fractions, that is, a ratio indicating the dose delivered above a specific LET‐threshold divided by the total delivered dose. In this study, three different LET‐thresholds were chosen: 3, 7 and 10 keV/μm, together with track and dose averaged versions of $Q_{\mathrm{eff}}$ and LET including all protons.

### 2.4 Benchmarking models

The models were benchmarked by the method outlined in our previous work,^31^ based on the “global fit” approach outlined by Abolfath et al.,^32^ with the performance of the novel dirty dose based models being compared to models based on ${\text{LET}}_{d}$ or $Q_{\mathrm{eff},d}$ as their radiation quality metric. The expression by which the ${\text{LET}}_{d}$ and $Q_{\mathrm{eff},d}$‐based models are fitted becomes:

(24) $$\ln\left(S\right)=-\alpha D-\beta D^{2}\approx -\alpha_{x}\left(R_{0}+t\frac{q}{\alpha_{x}/\beta_{x}}\right)D-\beta_{x}D^{2}$$

where q is the radiation quality metric used (${\text{LET}}_{d,\mathrm{protons}}$ or $Q_{\mathrm{eff},d,\mathrm{protons}}$ in this case), t is the fitting parameter and $R_{0}$ is the RBE for low‐LET proton dose. For the dirty dose based models using a single threshold value, the following expression is used, from Equation 3:

(25) $$\begin{array}{ccc} \ln(S) & = & -\alpha D-\beta D^{2}\\ & & \approx -\alpha_{x}\left(R_{0}\left(1-f\right)+{\hat{R}}_{D}\frac{f}{\alpha_{x}/\beta_{x}}\right)D-\beta_{x}D^{2} \end{array}$$

where ${\hat{R}}_{D}$ and f denote the free fitting parameter and dirty dose fraction, respectively. For two dirty dose thresholds, the following expression is instead applied

(26) $$\begin{array}{ccc} \ln(S) & = & -\alpha D-\beta D^{2}\\ & \approx & -\alpha_{x}\left(\;R_{0}\left(1-f_{\mathrm{low}}\;\mathrm{LET}\right)+\frac{{\hat{R}}_{1}\left(f_{\mathrm{low}}\;\mathrm{LET}-f_{\mathrm{high}}\;\mathrm{LET}\right)+{\hat{R}}_{2}f_{\mathrm{high}}\;\mathrm{LET}}{\alpha_{x}/\beta_{x}}\;\right)\\ & & D-\beta_{x}D^{2} \end{array}$$

where ${\hat{R}}_{1}$ and ${\hat{R}}_{2}$ and $f_{\mathrm{low}}\;\mathrm{LET}$ and $f_{\mathrm{high}}\;\mathrm{LET}$ denote the free fitting parameters and dirty dose fractions for the lower and higher dirty dose thresholds, respectively, as previously introduced.

For all models used, the $R_{0}$ value is constrained so that it cannot be smaller than 1. For the second dirty dose‐based model, the following constraint also applies:

(27) $$1\leq R_{0}\leq \frac{{\hat{R}}_{1}}{\alpha_{x}/\beta_{x}}\leq \frac{{\hat{R}}_{2}}{\alpha_{x}/\beta_{x}}$$

To ensure no decrease in RBE with increasing dirty dose fractions.

**FIGURE 2:**
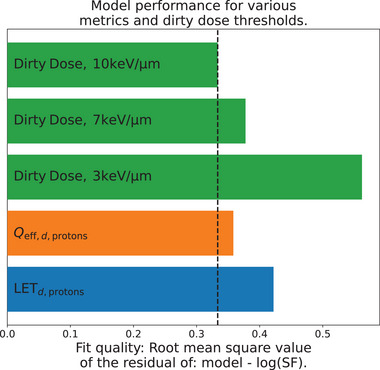
Shows the performance of different fitted RBE‐models, as measured by the root‐mean‐squared‐error of the fits to surviving fraction of each dose, cell and ${\text{LET}}_{d}$ combination. The dirty dose bars correspond to single‐threshold models, but the dual threshold models perform almost identically. LET, linear energy transfer; RBE, relative biological effectiveness.

The data is weighted based on the number of replicates for each dose‐${\text{LET}}_{d,\mathrm{primary}}$ combination. A simultaneous least‐squares fit is then conducted for all cell lines and dose levels, following conversion to logarithmic values of the surviving fraction. This approach aligns with the methodology outlined in Abolfath et al., 2017,^32^ also utilized in ref. [31]. The lmfit library in Python is employed, utilizing the “differential evolution” fitting method. The root‐mean‐squared‐error (RMSE) of the predicted and actual logarithmic survival fraction values serves as a benchmark to evaluate the performance of different fits. The resulting RBE‐weighted dose from a dirty dose‐based model with a single LET threshold of 7 keV/μm (using Equation 25) is shown in Figure 2 together with a fitted ${\text{LET}}_{d}$‐based model (using Equation 24). As a comparison, the RBE‐weighted dose from the McNamara model^10^ is also shown.

## 3 RESULTS

For all evaluated models, the fitted values of $R_{0}$ were always set to the lowest allowed value of 1. This lends support to the ansatz of the DDK model, namely that the clean dose component can be considered to be photon like. For the dual dirty dose‐based model, the fitted parameter ${\hat{R}}_{1}$ was also set to the lowest allowed value, meaning that the RBE value from dirty dose below the lowest threshold was set to the same as the RBE for clean dose, putting all the models weight on the high threshold parameter ${\hat{R}}_{2}$. This also means that the two different dirty dose models (with a single or two different thresholds) result in almost the same effective RBE model if a high dirty dose threshold is used in the single‐threshold model. This implies that, at least for this dataset, the very high LET values play the biggest role in determining the proton RBE value. For this reason, the high LET single threshold model actually performed very marginally better than the two‐threshold models, as the boundary constraints applied by Equation 27, forces the $R_{1}$ parameter to be larger than $R_{0}$ for some $\alpha_{x}/\beta_{x}$‐values; for the single threshold models, all LET values below the threshold always be scaled with the clean dose $R_{0}$ parameter. The obtained fitting parameters for all models are displayed in Table 1.

**TABLE 1:** Shows the obtained fitting parameters for all tested models.

	Fitting parameters				
Radiation quality metric(s)	$R_{0}$	t ^a^	${\hat{R}}_{D}$ ^b^	${\hat{R}}_{1}$ ^c^	${\hat{R}}_{2}$
${\text{LET}}_{d,\mathrm{protons}}$	1.00	4.78E‐1 m/keV·Gy	—	—	—
$Q_{\mathrm{eff},d,\mathrm{protons}}$	1.00	2.64E‐2 Gy	—	—	—
Dirty dose, ${\mathrm{LET}}_{\mathrm{DD}}$=3 keV/μm	1.00	—	6.73 Gy	—	—
Dirty dose, ${\mathrm{LET}}_{\mathrm{DD}}$=7 keV/μm	1.00	—	14.0 Gy	—	—
Dirty dose, ${\mathrm{LET}}_{\mathrm{DD}}$=10 keV/μm	1.00	—	20.3 Gy	—	—
Dirty dose, ${\mathrm{LET}}_{\mathrm{DD},\mathrm{low}}$=3 and ${\mathrm{LET}}_{\mathrm{DD},\mathrm{high}}$=7 keV/μm	1.00	—	—	2.87 Gy	13.8
Dirty dose, ${\mathrm{LET}}_{\mathrm{DD},\mathrm{low}}$=3 and ${\mathrm{LET}}_{\mathrm{DD},\mathrm{high}}$=10 keV/μm	1.00	—	—	2.87 Gy	19.8

^a^ For ${\mathrm{LET}}_{d,\mathrm{protons}}$‐ and $Q_{\mathrm{eff},d,\mathrm{protons}}$‐based models, see Equation 24.

^b^ For single threshold DDK‐models, see Equation 25.

^c^ For dual threshold DDK‐models, see Equation 26.

Abbreviations: DD, dirty dose; DDK, dirty dose kill; LET, linear energy transfer.

## 4 DISCUSSION

As discussed in more detail in previous work,^17^ utilizing in vitro data from a single large dataset is associated with both advantages in terms of minimizing random errors, but might similarly introduce systematic errors possibly biasing the results. However, since this study primarily focuses on conducting comparative analyses rather than aiming for immediate clinical applicability, a single high quality dataset with clearly described experimental setups, facilitating Monte Carlo re‐simulation for acquiring underlying particle spectra is optimal in this case.

Calculations of dirty dose offer some significant advantages compared to more commonly used averaged LET calculations. By focusing solely on specific LET thresholds, the process only involves determining whether a particle falls above or below these thresholds. This eliminates the need for end‐of‐range calculations where the proton LET values rapidly increase with minimal decreases in energy. Indeed, after the kinetic energies corresponding to the LET threshold values are estimated, no additional LET calculations are required; it suffices to determine whether dose deposition occurs above or below these specific kinetic energies.

The performance of the different models is shown in Figure 2. Dirty dose‐based models perform on par, or better, than ${\text{LET}}_{d}$ or $Q_{\mathrm{eff},d}$‐based models, while models using a lower threshold of 3 keV/μm perform significantly worse in comparison. The main reason for the bad performance of dirty dose models using only a lower 3 keV/μm threshold is that the dirty dose fraction saturates to unity early in the distal edge, as shown in the lower right panel of Figure 1. Since the data set used contains measurements from various locations in the distal edge with generally increasing RBE, this means that multiple different RBE values will share the same value of the selected radiation quality metric if the saturation occurs too soon, forcing the model to predict the same RBE value in the whole distal edge. When the threshold is increased to for example, 7 keV/μm, a saturation never occurs, and a significantly improved model performance is observed.

Dirty dose and track‐end based models share many similarities in their approach. Both utilize non‐averaged radiation quality metrics, simplifying calculations by focusing solely on what happens to the protons at the very end of their range. As the dirty dose threshold is increased, the residual range of the protons correspondingly shrinks. For instance, a proton with LET values of 3, 7, 10, and 15 keV/μm has ranges of 3, 0.5, 0.2 mm, and less than 0.1 mm, respectively. Consequently, adjusting the high LET dirty dose threshold after approximately 7 keV/μm would result in the dirty dose being placed within nearly identical voxels. Any decrease in the magnitude of the dirty dose due to a higher threshold therefore can be almost entirely offset by a larger fitting parameter, such as ${\hat{R}}_{D}$ or ${\hat{R}}_{2}$ in Equation 25 or 26, ensuring that the effective RBE model remains essentially unchanged.

Similarly, a track‐end based model becomes nearly equivalent to a dirty dose‐based model using a high LET threshold as the residual range approaches zero, and a track‐end based model would perform almost identically to the dirty dose model using the LET threshold of 10 keV/μm in Figure 2. Despite these similarities, dirty dose‐based models offer certain advantages. They eliminate the need to introduce non‐physical concepts such as “additional pseudo‐energy deposited by each track‐end” and avoids the necessity to divide by the mass of a voxel, as the dirty dose fraction remains invariant with respect to voxel size. As the DDK approach operates in the dose domain, applying it to for example, the LQ model becomes more straightforward. Furthermore, in a dirty dose‐based approach the increase in RBE does not stem from the track‐ends themselves, but from the elevated LET values of the protons just before they stop, in line with the proposed biological mechanisms underlying the elevated RBE values.^33^ For example, if a proton enters a voxel with extremely low energy and immediately stops, and thereby deposits almost no dose, a track‐end based model would put all the elevated RBE in the same voxel the proton stopped in, whereas a dirty dose model would put most of the elevated RBE in the voxel superseding it (where most of the high LET dose was deposited).

The exact chosen LET values for the dirty dose threshold are not explicitly justified in this work. However, as discussed above, the choice of the higher threshold will have almost no practical difference due to the short remaining proton range, and could possibly be set anywhere in the range of 7‐15 keV/μm. However, due to difficulties in creating experimental setups for DDK conditions, a threshold in the lower part of this range might be optimal, maybe in the range of 7‐10 keV/μm. Regarding the possible lower LET threshold, 3 keV/μm would correspond to a proton residual range of a typical clinical voxel size, which is practically convenient. It might also be the case that a lower threshold shall not be used altogether, as the results from this in vitro dataset would suggest.

Compared to ${\text{LET}}_{d}$‐based models, models based on dirty dose metrics typically predict a lower RBE in the entry region of a Bragg peak (see the two lower panels of Figure 1), but catches up in the distal edge. This is a result of almost no dose being delivered above the dirty dose threshold in the whole entry region, resulting in dirty dose fractions very close to zero. While ${\text{LET}}_{d}$ is still low in the same region, it is high enough that the RBE contribution is not completely negligible, thereby leading to a slightly increased RBE also in the entry region. For two opposing SOBP's, as shown in the upper right panel of Figure 1, both the ${\text{LET}}_{d}$‐based models from this dataset and the ${\text{LET}}_{d}$‐based McNamara model^10^ predict an RBE of about 1.14 in the center part, compared to 1.06 for the dirty dose‐based model using a single 7 keV/μm threshold. In the dose fall‐off region, as the dirty dose fraction sharply increases (compared to a smaller relative increase in ${\text{LET}}_{d}$), all three RBE models predict more similar RBE values. As a general trend, increased RBE from dirty dose‐based models are more concentrated around the distal edges of the dose distribution, whereas ${\text{LET}}_{d}$‐based models exhibit generally higher RBE values, with smaller RBE increases around the distal edges. It should be noted that the in vitro data which this study is based on generally also exhibit very low RBE values in the entry region with a sharper, non‐linear increase for higher ${\text{LET}}_{d}$‐values,^31^ in line with the dirty dose metrics.

For the vast majority of in vitro RBE experimental setups, a beam with a narrow LET‐spectrum is produced by the proton accelerator, which is then degraded by a range shifter to produce different LET spectra along which the RBE values vary. This results in a broadening of the LET spectra for the higher ${\text{LET}}_{d}$ measurements due to energy range straggling. One consequence of this is that it becomes difficult to construct a RBE(LET) function (for a single LET value, i.e., not an averaged value across the whole spectrum), and some kind of averaging, dirty dose, or track‐end approach is ultimately needed to utilize the produced data into a variable RBE model. It would, in our opinion, be very interesting to instead produce in vitro RBE data by having narrow LET‐spectra even for the lower proton energies. This way, a RBE(LET) or RBE(E) model can be produced, and an RBE value from a mixed field can then be calculated by weighing the RBE values of individual LET‐spectra segments by dose contribution. This would negate the need for averaging (which corresponds to assuming a linear RBE(LET) relationship), or other pre‐processing of the spectrum and would ultimately result in a purer RBE model with less assumptions being made.

As the residual range of the ${\mathrm{LET}}_{\mathrm{DD},\mathrm{high}}$ protons is extremely short, the voxel distribution of protons with energies corresponding to the threshold value will be virtually the same as the distribution of the track‐ends. Hence, there is no practical need to know the RBE(LET) relationship in this LET region; an average RBE value determined by measuring $\xi^{\prime }$ is sufficient. Performing measurements utilizing this “DDK‐setup” therefor has the potential to create better “bang for the buck” models compared to traditional microscopic setups, as either less total number of measurements need to be performed or smaller statistical uncertainties can be obtained by concentrating the same total number of measurements to fewer setups.

## 5 CONCLUSION

This work introduces a new concept for RBE modeling aimed specifically at treatment planning. We demonstrate that DDK model parameters can be determined from “classical” in vitro clonogenic cell survival assay data, with the caveat that accuracy is hampered by intrinsic limitations imposed by ill conditioned measurement situations. Nevertheless, for the used data set, models based on the dirty dose concept performed equally well or better compared to conventional radiation quality metrics such as ${\text{LET}}_{d}$ and $Q_{\mathrm{eff},d}$, while offering advantages both with respect to calculation robustness and measurement techniques. A point worth noticing here is that the data set was measured at a single beam energy and produced cell survival data only valid for exactly that irradiation arrangement. Through the DDK model formalism, one can extract cell line specific parameters from such data which allows generalization to, in principle, any irradiation situation. Finally, we propose a novel cell survival assay approach in which survival is measured directly at DDK or near DDK conditions. This entails a new type of measurement methodology to be implemented with an increased precision and a large reduction in the number of survival data points needed per cell line.

## CONFLICT OF INTEREST STATEMENT

The authors declare no conflicts of interest.

## Footnotes

1 Throughout the paper, when we write “LET” we mean the linear energy transfer of a single particle. Any other derived values obtained by for example, averaging is explicitly denoted by a suffix as in for example, ${\text{LET}}_{d}$.

^**^
Note that in this work $D_{D}$ and $D_{C}$ denote the physical dose of respective dose component. This is in contrast to Heuschel et al.^18^ where an RBE = 1.1 factor was included in the $D_{D}$ and $D_{C}$ dose components.
